# A comprehensive model for characterizing Chinese female facial skin laxity by expert assessment, consumer perception, and noninvasive skin physiological parameters

**DOI:** 10.1038/s41598-025-19750-9

**Published:** 2025-10-14

**Authors:** Yusha Zi, Jiaqi Zhang, Qi Liu, Sisi Chang, Wei Liu, Hua Zhao, Jianwei Liu

**Affiliations:** 1 Amway (Shanghai) Technology Development Co., Ltd., Shanghai, 201203 China; 2 Department of Cosmetics, School of Light Industry Science and Engineering, Beijing Technology and Business University, Beijing, 100048 China; 3 Beijing EWISH Testing Technology Co., Ltd., Beijing, 100142 China; 4 Air Force Medical Center, Department of Dermatology, People’s Liberation Army, Beijing, 100142 China

**Keywords:** Facial skin laxity, Computer modelling, Two-alternative forced choice, Subjective evaluation, Engineering, Health care, Mathematics and computing, Medical research

## Abstract

This study used expert assessment, consumer perception, and noninvasive measurements of skin physiological parameters to develop a mathematical model for characterizing facial skin laxity in Chinese females, providing the scientific basis for personalized beauty and skin-firming strategies. The study involved 64 Chinese females (18–60 years) whose facial regions were imaged using VISIA-CR. Five experts assessed facial skin laxity using VISIA-CR images, while 72 consumers compared paired images to identify higher laxity. Noninvasive measurements of skin elasticity and other parameters were taken. The data were analyzed using Bradley-Terry analysis and support vector machine regression modeling. Consistent with expectations, facial skin laxity was negatively correlated with skin elasticity R2 values and positively correlated with skin elasticity F4 values. A novel Facial Skin Laxity Index (*FSLI*) was introduced to evaluate product efficacy in reducing laxity. Facial skin laxity can be characterized and quantified using a combination of expert assessments, consumer perceptions, and noninvasive measurements. The *FSLI* enables precise evaluation of product performance in improving facial skin laxity.

## Introduction

Facial skin laxity is the result of a combination of factors. First, with age, collagen and elastin fibers in the skin gradually decrease, leading to a loss of skin elasticity, which in turn triggers skin laxity^1–3^. In addition, increased exposure to ultraviolet (UV) rays accelerates facial wrinkles and sagging^4^. Other external factors, such as gravity and environmental pollution, can also negatively affect facial skin^5,6^. For Asians, the structural features of the face and the physiological properties of the skin determine the uniqueness of the signs of aging. For example, loss of volume in specific areas of the face may be an important factor contributing to their skin laxity^7^.

Facial skin laxity presents itself in multiple forms, typically characterized by under-eye bags, drooping eyelids, the deepening of facial lines, the emergence of perioral lines, and a blurred jawline definition^4,8–14^. While the specific manifestations can differ from person to person, collectively they contribute to a visage of aging, potentially impacting an individual’s appearance and self-esteem^15–19^. Research indicates that individuals in Asian countries are particularly attentive to skin firmness and are more likely to seek interventions to combat aging signs at an earlier stage compared to their Western counterparts^20^. In addition, aesthetic standards differ due to cultural backgrounds. East Asian cultures may place more emphasis on the firmness and smoothness of the overall facial contours, while Western cultures may emphasize the three-dimensionality of facial features and natural aging. In East Asia, where lighter nasolabial folds are often considered a sign of youth^21^, wrinkles and aging in the mid-face region can cause the perceived age of the skin to differ from its actual age^22^. Therefore, East Asians may take measures to reduce the depth of nasolabial folds by injecting fillers or undergoing laser treatments^23,24^. In the West, people may be more inclined to accept the natural presence of nasolabial folds as part of facial expression rather than a mere sign of aging^25^. Hence, more race-specific factors need to be considered when dealing with Asian facial aesthetics^16,26–28^.

Current research evaluating facial skin laxity can be broadly classified into three categories: clinical assessment, imaging analysis, and the measurement of objective skin physiological parameters^10,12,29–34^. Clinical assessment serves as the fundamental and widely employed method for evaluating facial skin laxity. It primarily depends on the professional expertise and observational skills of the assessor, who typically employs a grading system ranging from grade I (mild) to grade IV (severe) to characterize the severity of skin laxity^35^. In addition to this grading system, various questionnaires and scales have been developed for clinical use^36^, including the validated *Fitzpatrick Wrinkle, Fold, and Tissue Laxity Scale* (FWFTLS)^37^, the *Facial and Neck Laxity Grading Scale* (FLR)^38^, and several others. Clinical assessment methods, while more intuitive, are inherently subjective and can vary between assessors, which may compromise their consistency and reliability. In contrast, imaging analysis and the measurement of objective skin physiological parameters offer a more objective and precise approach to evaluation. Common imaging techniques include ultrasound imaging and 3D imaging. Ultrasound imaging provides detailed visualization of structural changes within the skin and underlying tissues, such as the loosening of collagen fibers and the distribution of adipose tissue^33,39^. 3D imaging utilizes sophisticated software to create detailed three-dimensional representations of facial skin, facilitating easy comparison of changes over time and supplying quantitative data for analysis^31^. The Cutometer is a widely utilized device for assessing skin laxity by quantifying the skin’s elasticity and resilience. Furthermore, elastography, a method that employs ultrasound or magnetic resonance imaging (MRI) to measure the skin and dermis’s modulus of elasticity, offers a quantitative measure of skin laxity^40^. These advanced tools help mitigate subjective errors, enhancing the precision and reproducibility of skin laxity assessments. In conclusion, the various methods for assessing facial skin laxity each possess distinct attributes. Clinical assessments offer intuitive yet subjective insights, whereas imaging analyses and quantitative measurement tools deliver a more objective and accurate means of evaluation, providing robust data support.

However, these tools have not been fully and rigorously characterized or modeled, particularly within the context of the Chinese female population. For a comprehensive and precise evaluation of facial skin laxity, it is essential to consider employing a combination of assessment methods^41^. This study employed a comprehensive approach, integrating expert assessments, consumer perceptions, and noninvasive measurements of skin physiological parameters, to develop a mathematical model that characterizes facial skin laxity in Chinese females. This model establishes a scientific foundation for the development of personalized beauty and skin-firming strategies.

## Materials and methods

### Participants and facility

The participants of this study were healthy Chinese females aged 18-60 years old and had to meet the following conditions: (1) those who are troubled by facial skin laxity; (2) self-reported wrinkles or fine lines around the eyes and laxity; (3) the presence of pronounced facial wrinkles or signs of sagging was confirmed by a professional assessor; (4) those who are able to understand the trial process and sign an informed consent form.

The study complies with the *Declaration of Helsinki* and has been authorized by the participants’ portrait rights and informed consent forms (ICF). The study was conducted at Beijing EWISH Testing Technology Co., Ltd. in Beijing, China. All participants in the study were asked to clean their facial skin with the same cleanser in a laboratory setting and were acclimatized to constant temperature (21 ± 1°C) and humidity (50 ± 10%) for 30 min before image and data acquisition. A total of 142 participants who met the screening requirements had their facial images and skin physiological parameters collected during the study.

### Image capture

Front, left, and right facial images of the participants were acquired using the VISIA-CR (Canfield Scientific Inc., Parsippany, NJ, USA), and the light sources of the acquired images consisted of standard light 1, standard light 2, parallel-polarized light, cross-polarized light, and UV light.

### Noninvasive measurement of skin physiological parameters

The noninvasive physiological parameters measured in this study and their testing instruments and sites can be seen in Table 1.

**Table 1:** Non-invasive physiological parameters and their testing instruments and sites.

Non-invasive physiological parameter	Instrument	Site
Hydration (H)	Corneometer CM825, (Couragc + Khazaka Elcctronic, Kǒln, NRW, Germany)	Either cheek
Transepidermal water loss (TEWL)	Tewameter TM 300, (Couragc + Khazaka Elcctronic, Kǒln, NRW, Germany)	Either cheek
Skin glossiness (SG)	Glossymeter GL200, (Couragc + Khazaka Elcctronic, Kǒln, NRW, Germany)	Either cheek
Collagen content at 2 mm of skin (CC2)	SIAMETRICS SIAscope V (MedX Health Corp., Mississauga, Ontario, Canada)	Either cheek
Advanced glycation end products (AGEs)	AGE Reader (Diagnoptics Technologies B.V., Groningen, Netherlands)	Either cheek
Dermis thickness (DT) and dermis density (DD)	Ultrasound & DermaLab® Series SkinLab Combo (Cortex Technology ApS, Aalborg, Mariagerfjord Municipality, Denmark)	Either cheek
Skin elasticity (F3, F4, Q1, R2, R5 value)	Cutometer MPA580, (Couragc + Khazaka Elcctronic GmbH, Kǒln, NRW, Germany)	Either cheek
Perimeter, volume, and depth of underneath eye wrinkles (UEW_P, UEW_V, UEW_D)	Derma TOP (EO-TECH SAS, Marcoussis, IDF, French)	Underneath eye wrinkles on either side
Area, volume, and depth of marionette lines (ML_A, ML_V, ML_D)	Derma TOP (EO-TECH SAS, Marcoussis, IDF, French)	Marionette lines on either side
Area, volume, and depth of nasolabial fold (NF_A, NF_V, NF_D)	Derma TOP (EO-TECH SAS, Marcoussis, IDF, French)	Nasolabial fold on either side
Dermal–epidermal junction (DEJ)	SUPERVISION780 (Beijing Transcend Vivoscope Bio-Technology Co., LTD., Beijing, China)	Either cheek
Epidermal thickness (ET)	SUPERVISION780 (Beijing Transcend Vivoscope Bio-Technology Co., LTD., Beijing, China)	Either cheek

**Fig. 1:**
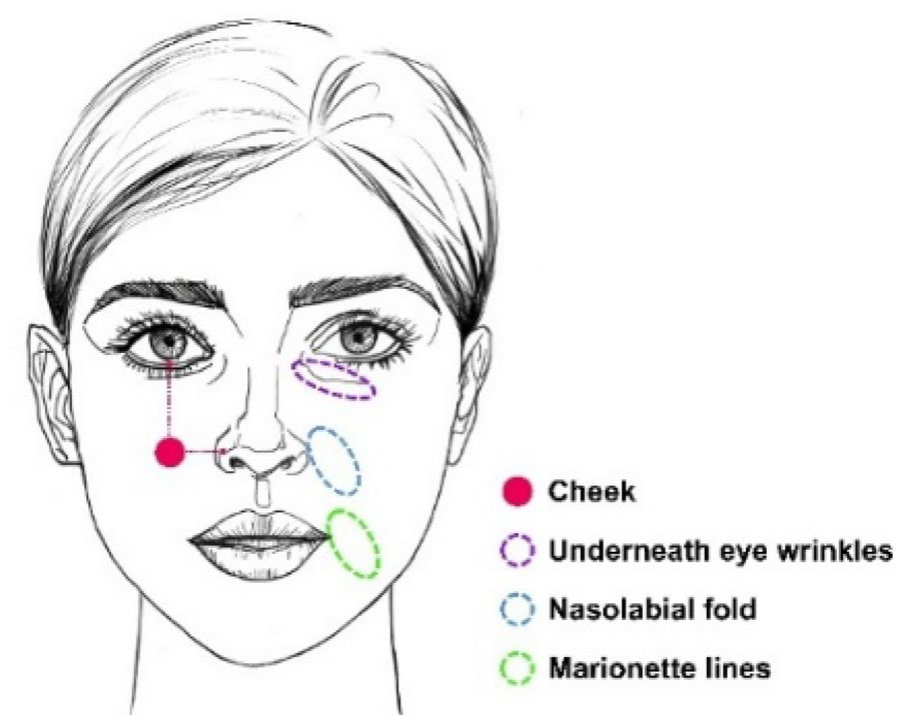
Schematic diagram of the test sites.

Non-invasive physiological parameters of the cheek, underneath eye wrinkles, nasolabial fold, and marionette lines on either side of the participants were collected according to a randomized table. The test site of the cheek is the intersection of the vertical line extending downward from the middle of the eye and the horizontal line at the ala nasi, and a schematic diagram of the specific test site can be seen in Fig. 1.

### Expert assessment

Five experienced professional evaluators estimated apparent age from front VISIA-CR images taken under standard light 1 of 142 healthy Chinese women aged 18-60. Apparent age assessment requires a combination of facial skin pigmentation, skin tone, wrinkles, contour, and other dimensions of age-stage skin characteristics, and the assessment data were recorded in whole numbers. After the initial, independent, blinded evaluations, the panel convened to reach a consensus, and the resulting value was recorded as the definitive expert assessment.

Furthermore, the same five evaluators screened the front VISIA-CR images acquired under standard light 1 for facial skin laxity in all 142 participants to identify suitable candidates for the subsequent consumer perception study. Five evaluators were required to exclude the effects of skin pigmentation and skin tone, focusing on the degree of visibility of underneath eye wrinkles, nasolabial folds, and marionette lines on both sides of the face, and the degree of cheek depressions and jawline edge definition. Finally, 64 participants were selected for the consumer perception study. Participants should be selected to cover a wide range of facial skin laxity, and the study should be conducted within a manageable range. The 64 participants were labeled equally into two categories, “more facial skin laxity” and “less facial skin laxity,” by the subjective assessment of five professional evaluators.

### Consumer perception

The study evaluated the VISIA-CR images taken under standard light 1 of 64 participants for consumer perception by a pairwise comparison test, also known as a two-alternative forced choice (2-AFC) test. 64 participants were randomly divided into eight groups, each of which consisted of four people labeled as having “more facial skin laxity” and four people labeled as having “less facial skin laxity.” To improve perceptual accuracy, we followed the Thurstonian modeling framework^42–44^ and comprehensively paired participants labeled as “more facial skin laxity” (M1, M2, M3, and M4) with participants labeled as “less facial skin laxity” (L1, L2, L3, and L4) in each 8-person group, resulting in 16 unduplicated pairs (shown in Table 2).

**Table 2:** Examples of participant matching programs.

Pairs	L1	L2	L3	L4
M1	M1 & L1	M1 & L2	M1 & L3	M1 & L4
M2	M2 & L1	M2 & L2	M2 & L3	M2 & L4
M3	M3 & L1	M3 & L2	M3 & L3	M3 & L4
M4	M4 & L1	M4 & L2	M4 & L3	M4 & L4

**Fig. 2:**
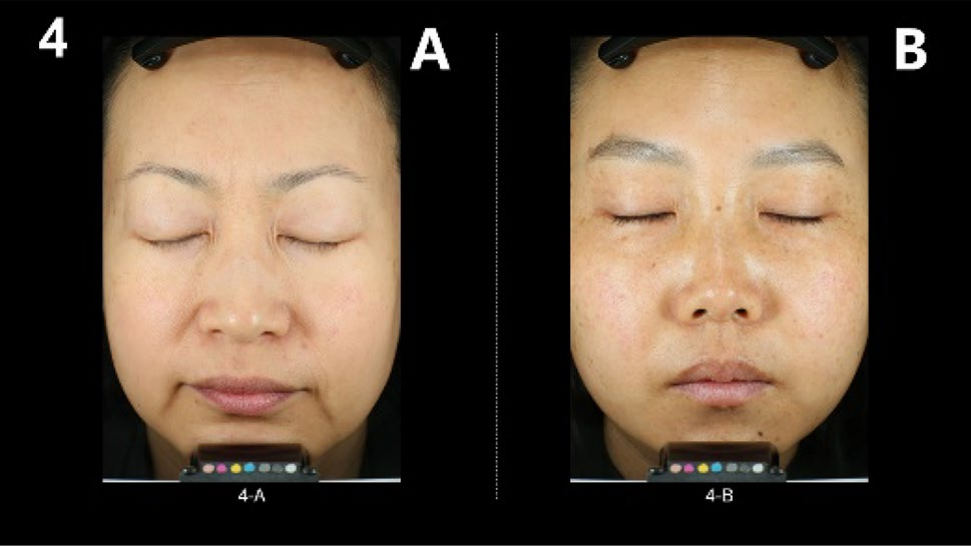
Example of consumer perception atlas for facial skin laxity.

In order to increase the perceptual accuracy, one exchange of position was considered, so there are 32 pairs per group, totaling 256 pairs for 8 groups. A consumer perception atlas for facial skin laxity was created by randomly disrupting the order of each pair, the form of which can be seen in Fig. 2. Consumers will choose between 256 A and B pairs for “more facial skin laxity.”

**Fig. 3:**
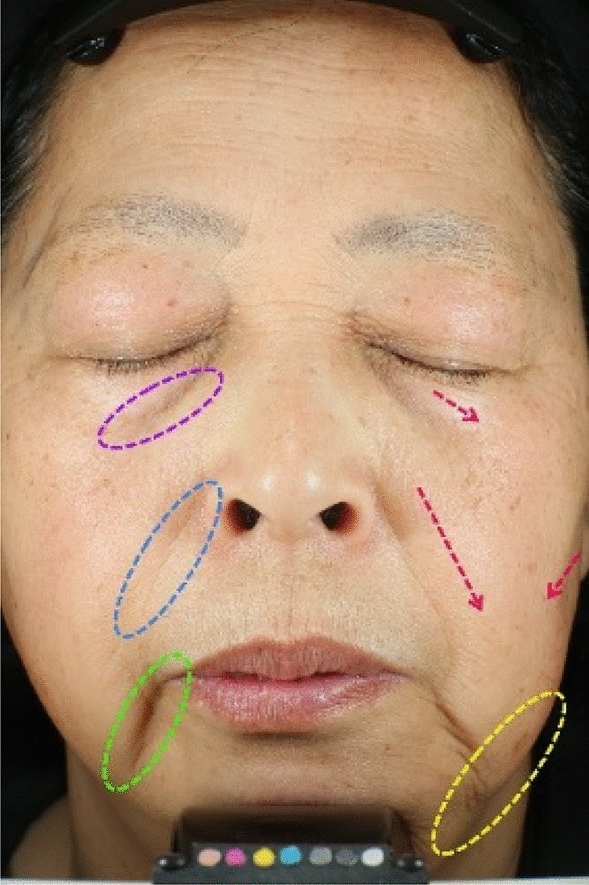
Schematic diagram of consumer perception training for facial skin laxity.

Before consumer perception, participants were provided with a brief training session on the concept and manifestations of facial skin laxity, the objectives of their perception, and the data recording procedures. This study gathered insights from 11 cosmetic industry professionals regarding their understanding of facial skin laxity, which can be encapsulated as follows: Facial skin laxity denotes the diminished elasticity and firmness of the facial skin attributable to some factor, leading to visible sagging, looseness, and loss of smooth contours. This condition is characterized by specific signs such as drooping eye bags, pronounced eye wrinkles, deepening nasolabial folds, the formation of marionette lines, sagging apple muscles, cheek hollowing, and an indistinct jawline, as illustrated in Fig. 3.

Consumers evaluated the front images of the paired VISIA-CR standard light 1 images in the 2-AFC test, selecting the “more facial skin laxity” from each pair of participants. In this study, it was only necessary to assess the side of A and B that had more facial skin laxity, not to determine whether there was a difference between A and B. Therefore, the statistical method of pairwise comparisons with a one-sided test was chosen to calculate the number of consumers required^45^. The minimum number of evaluators *n* is calculated from the normal approximation of the binomial distribution, as illustrated in Eq. (1). At least 68 evaluators are needed for the one-sided pairwise test under the conditions of *α *= 0.05, *β *= 0.5, and *P*_*d *_= 20% (*α*: alpha-risk, false-positive rate; *β*: beta-risk, false-negative rate; *P*_*d*_: percentage of evaluators who detect a discrepancy)^46^.

1 $$n \approx \frac{{z_{1 - \alpha } \sqrt {p_{0} 1 - p_{0} } + z_{1 - \beta } \sqrt {p_{1} 1 - p_{1} } }}{{p_{1} - p_{0} }}^{2}$$

${p}_{0}=0.5$, the probability of correct choice when there is no significant difference;

${p}_{1}={p}_{d}+(1-{p}_{d})\times 0.5=0.2+(1-0.2)\times 0.5=0.6$, the probability of correct choice when there is significant difference; ${z}_{1-\alpha }=1.64$ (α = 0.05); ${z}_{1-\beta }=0$ (*β* = 0.5); *n *≈ 67.24.

In this study, a total of 72 participants were recruited to partake in consumer perception assessments, comprising 36 individuals with professional backgrounds in cosmetics and 36 without such expertise.

In summary, the decisions made by the 72 participants reflect the outcomes of their consumer perceptions. The study evaluated a total of 64 participant facial images, which encompassed 256 pairwise comparison events, ultimately yielding 18,432 responses in total.

### Quantification of consumer perception results

The analysis of consumer responses involved calculating the odds ratio for selecting *T*, which represents “more facial skin laxity,” in each pair comparison. Subsequently, the logit function was applied to these odds ratios (denoted as Logit*[T]*), as illustrated in Eq. (2).

2 $${\text{Logit}}\left( T \right) = \ln \left( {\frac{{P_{T} }}{{1 - P_{T} }}} \right)$$

where *p* denotes the binomial probability and $\frac{{P_{T} }}{{1 - P_{T} }}$ signifies the odds ratio of selecting *T*. This method facilitates the transformation of consumer responses, which are in binary format, into a dataset that follows a normal distribution. This conversion allows for the mapping of the probability of consumer choices across a continuum from negative infinity to positive infinity^47^. Out of the 18,432 responses collected in the pairwise comparison study, the normal order and reversed order responses were averaged to generate 128 unique high-low pairs across the 64 study participants.

### Modelling

Correlations between expert apparent age assessment results, consumer perception differences, and the combined effect of noninvasive skin physiological parameters were established by the support vector machine (SVM) regression model. Consumer-perceived results demonstrate selection for “more facial skin laxity” in 128 unduplicated pairs. Quantifying consumer perception through Logit(*T*).

**Fig. 4:**
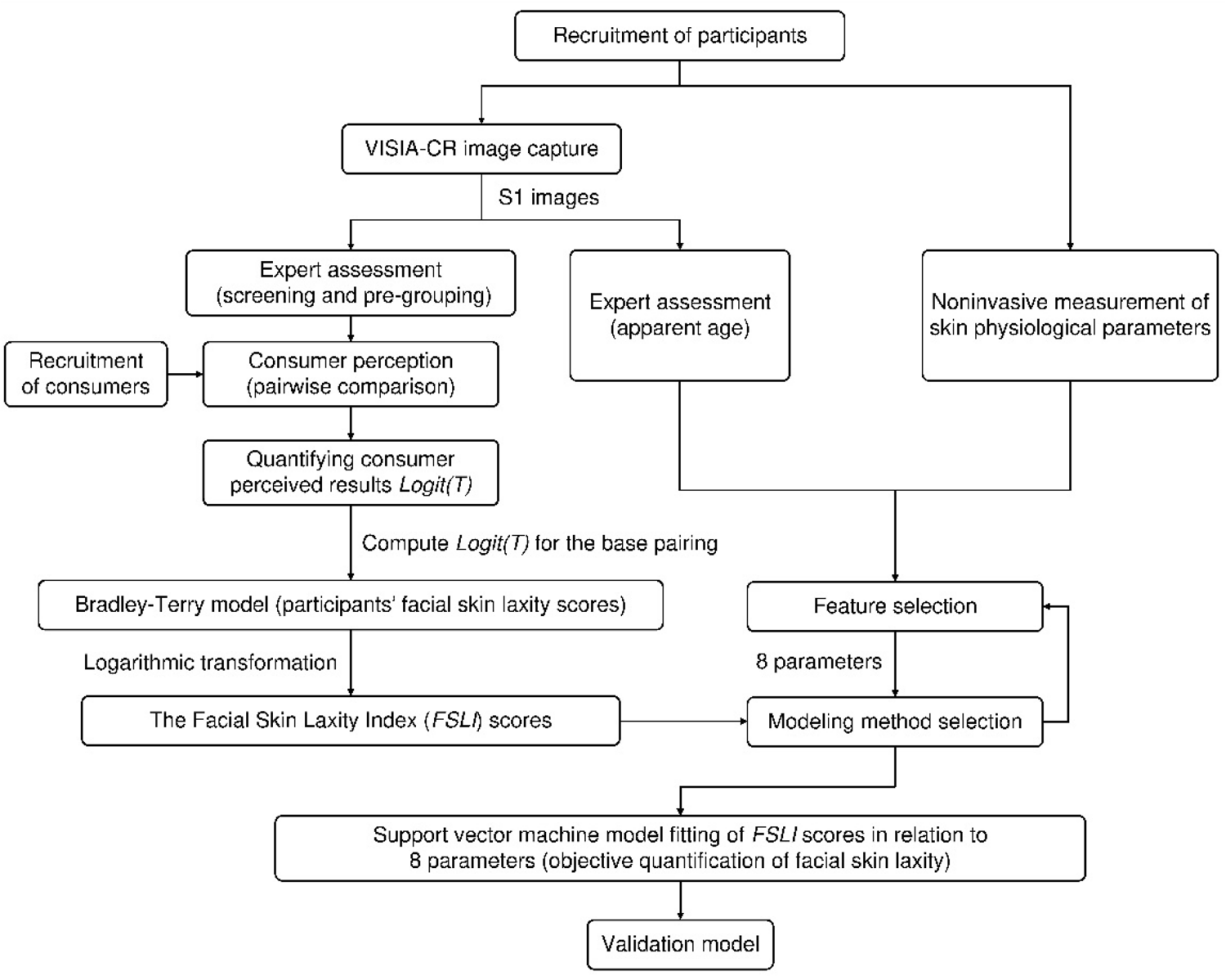
Technical roadmap for modeling the *FSLI*.

The Bradley-Terry model, a probabilistic model that allows for combining and estimating the results of pairwise comparison experiments^44^, was used to convert consumer-perceived Logit(*T*) results of facial skin laxity for 64 participants into a latent facial skin laxity score. In accordance with the Bradley-Terry scoring matrix, we integrated the raw Logit(*T*) scores obtained directly from consumers’ perceptions and applied a sigmoid function to the entire set of Logit(*T*) results. By inputting the results into the Bradley-Terry model, we derived a Bradley-Terry score for facial skin laxity among 64 participants. Subsequently, a linear relationship between the degree of facial skin laxity score and expert assessment results and objective parameters was established. See Fig. 4.

## Results

### Distribution of noninvasive skin physiological parameters

A comprehensive raw dataset encompassing facial skin parameters was gathered from 142 participants, comprising a total of 24 parameters. This dataset included expert assessments of apparent age along with 23 noninvasive physiological skin measurements, as shown in Table 3. The Shapiro-Wilk normality test showed that some of the parameters were normally distributed and some were not normally distributed.

**Table 3:** Distribution properties of acquisition parameters.

Parameters*	Mean ± SD	W-value	*p*-value
H (C.U.)	33.323 ± 6.547	0.972	0.154
TEWL (g/(h·m^2^))	12.389 ± 3.569	0.883	0.000
SG (dimensionless)	4.844 ± 0.611	0.978	0.317
CC2 (dimensionless)	130.693 ± 20.817	0.587	0.000
AGEs (AU)	2.393 ± 0.625	0.967	0.082
DT (μm)	1575.047 ± 214.109	0.986	0.664
DD (dimensionless)	13.230% ± 2.116%	0.982	0.466
F3 (dimensionless)	5.006 ± 0.863	0.969	0.104
F4 (dimensionless)	11.965 ± 1.658	0.991	0.937
Q1 (dimensionless)	0.499 ± 0.462	0.98	0.387
R2 (dimensionless)	0.542 ± 0.046	0.978	0.325
R5 (dimensionless)	0.564 ± 0.054	0.965	0.066
UEW_P (mm)	72.200 ± 40.840	0.949	0.010
UEW_V (mm^3^)	0.339 ± 0.387	0.686	0.000
UEW_D (mm)	− 0.033 ± 0.015	0.616	0.000
ML_A (mm^2^)	8.354 ± 4.337	0.951	0.013
ML_V (mm^3^)	0.530 ± 0.471	0.839	0.000
ML_D (mm)	− 0.037 ± 0.018	0.608	0.000
NF_A (mm^2^)	6.962 ± 4.752	0.833	0.000
NF_V (mm^3^)	0.406 ± 0.401	0.742	0.000
NF_D (mm)	− 0.036 ± 0.012	0.833	0.000
DEJ (dimensionless)	3.191 ± 0.859	0.975	0.219
ET (μm)	42.704 ± 6.502	0.952	0.014
AA (years)	48.469 ± 7.464	0.921	0.001

*H* hydration, *TEWL* transepidermal water los, *SG* skin glossiness, *CC2* relative concentration of collagen in the skin at a depth of 2 mm, *AGEs* advanced glycation end product, *DT* dermis thickness, *DD* dermis density in %, *F3* area within the enveloped curve, represents the skin fatigue, *F4* area beneath the enveloped curve, represents the firmness of the skin and the resistance to the suction, *Q1* total recovery area, increases with higher elastic recovery^48^, *R2* visco-elasticity, resistance to the mechanical force versus ability of recovery, *R5* net elasticity, the ratio of the elastic part of the suction phase to the immediate recovery during the relaxation phase, *UEW_P* perimeter of underneath eye wrinkles, *UEW_V* volume of underneath eye wrinkles, *UEW_D* depth of underneath eye wrinkles, *ML_A* area of marionette lines, *ML_V* volume of marionette lines, *ML_D* depth of marionette lines, *NF_A* area of nasolabial fold, *NF_V* volume of nasolabial fold, *NF_D* depth of nasolabial fold, *DEJ* dermal-epidermal junction, ratio of the area of the dermal-epidermal interface to the area of the horizontal plane, *ET* epidermal thickness, *AA* apparent age.

**Fig. 5:**
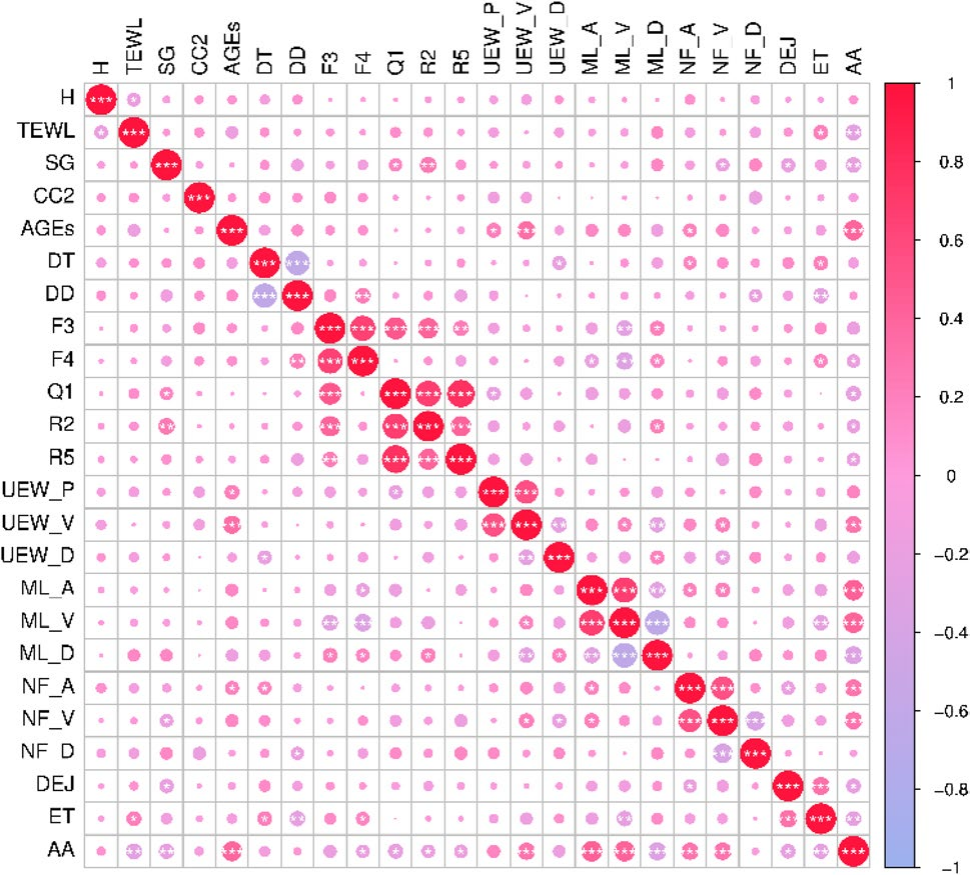
Acquisition parameter correlation.

Of the 24 parameters collected for the study, 2 parameters (skin elasticity F3 and R5 values) were excluded from the modeling process due to their direct correlation with skin elasticity R2 and F4 values, as shown in Fig. 5. The remaining 22 parameters were considered independent (variance inflation factor < 5) and represent skin attributes such as moisture, wrinkles, elasticity, and collagen.

### Consumer perception response

The results from the 256 pairs that contained swapped positions were integrated, yielding 128 unique pairs of consumer perceptions. A binomial test was conducted, and the results indicated that the consumer perceptions were significantly different across all pairs (*p *< 0.05, α = 0.05, two-tailed). This suggests that the consumer could significantly perceive the difference between 128 pairs of non-repeated images and choose the one with “more facial skin laxity” among them. The pairwise combinations used for consumer perceptions were grouped by expert assessment, therefore, Cohen’s kappa was used to analyze the consistency of the data from the 72 consumer assessments with the expert-assessed “standard answers.” It was found that the κ-mean of 72 consumers was 0.929 with a 95% confidence interval of [0.913, 0.945]. The κ-mean of 36 consumers with a professional background in cosmetics was 0.932 with a 95% confidence interval of [0.909, 0.956]. The κ-mean of 36 consumers without a professional background in cosmetics was 0.926 with a 95% confidence interval of [0.903, 0.949], indicating that there was a high degree of consistency between consumer perceptions and expert evaluations. Consumers with a professional background in cosmetics had a higher concordance with expert assessments compared to those without a professional background in cosmetics, but there was no significant difference between the two groups in terms of perceptions of facial skin laxity (*p *= 0.29 > 0.05, Mann-Whitney U test, α = 0.05, two-tailed).

The perceived outcomes for 128 unique pairs of participant atlases were analyzed and quantified utilizing the Logit*(T)* model. Participants exhibiting more pronounced differences in perceivable facial skin laxity corresponded to higher absolute |Logit*(T)|* values, with the reverse also being true. In this study, certain pairs demonstrated markedly greater differences in facial skin laxity, leading to instances where every consumer uniformly selected one face over another within those pairs. Consequently, this resulted in a slight skew in the distribution of Logit*(T)* values towards the higher end of the scale’s ratings.

### Modelling

The Bradley-Terry score was logarithmically transformed and named the Facial Skin Laxity Index (*FSLI*). The model was developed using SVM regression in R, establishing a relationship between the *FSLI* and the apparent age assessment results, as well as the noninvasive skin physiological parameters of 64 participants. Out of the 22 parameters considered in the modeling process, 8 were ultimately selected to ensure the model’s accuracy. The outcomes of the modeling are presented in Table 4.

**Table 4:** R modeling results.

Parameters composition	Number of parameters	R^2^	RMSE
CC2, F4, Q1, R2, UEW_V, ML_V, NF_V, ET	8	0.811	0.0518

R2: 0.811, the coefficient of determination, measures a model’s capacity to explain the variance in the target variable; the closer the value is to 1, the better the model fits the data. RMSE: 0.0518, the root mean square error, represents the average magnitude of the errors between predicted and actual values, indicating the degree of deviation; the closer the value is to 1, the higher the prediction accuracy of the model. Higher values of the *FSLI* represent a higher degree of facial skin laxity.

**Fig. 6:**
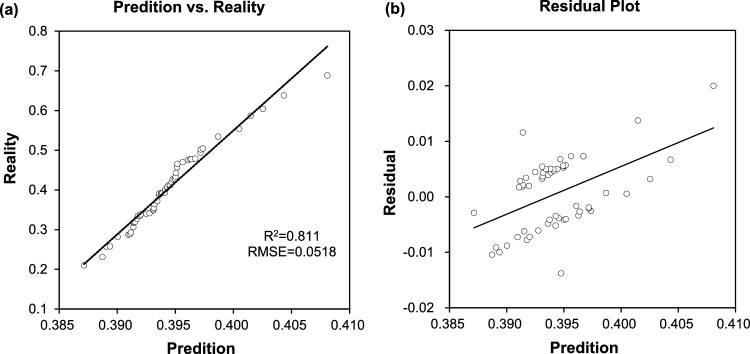
Model performance. (**a**) Predicted vs. reality *FSLI* scores; (**b**) Residual plot.

Correlation analysis was done between the *FSLI* values predicted by the model and the actual values, and the correlation coefficient between the actual values and the predicted values was 0.433, *p *< 0.001, which showed that there was a significant correlation between the actual values and the predicted values, indicating that the model was well fitted. Fig. 6 shows the predicted vs. reality *FSLI* scores (Fig. 6a) and residual plot (Fig. 6b) of the *FSLI* mode.

### Validation of *FSLI* model

**Fig. 7:**
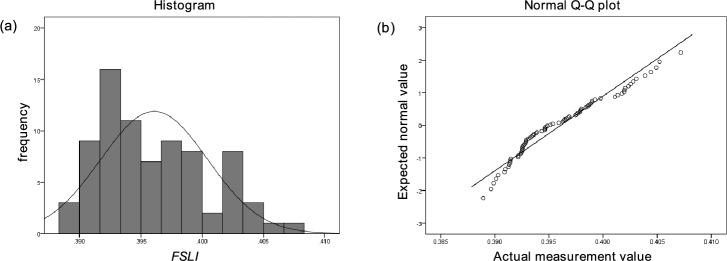
Distribution of facial skin laxity index (*FSLI*) in 78 Chinese female. (**a**) Histogram; (**b**) Q-Q plot.

The *FSLI* model described above was applied to measure facial skin laxity in 78 Chinese female who were not previously involved in modeling among the 142 participants in this study, and *FSLI* scores were calculated. Fig. 7 shows the histogram (Fig. 7a) and Q-Q plot (Fig. 7b) of the measured *FSLI* values, However, the distribution of *FSLI* in this population is non-normal (*p *= 0.004, Shapiro-Wilk normality test), which may be attributed to the relatively small sample size. The mean of this distribution is 0.3961, the median is 0.3950, and the standard deviation is 0.00436.

### Application of *FSLI* model

**Fig. 8:**
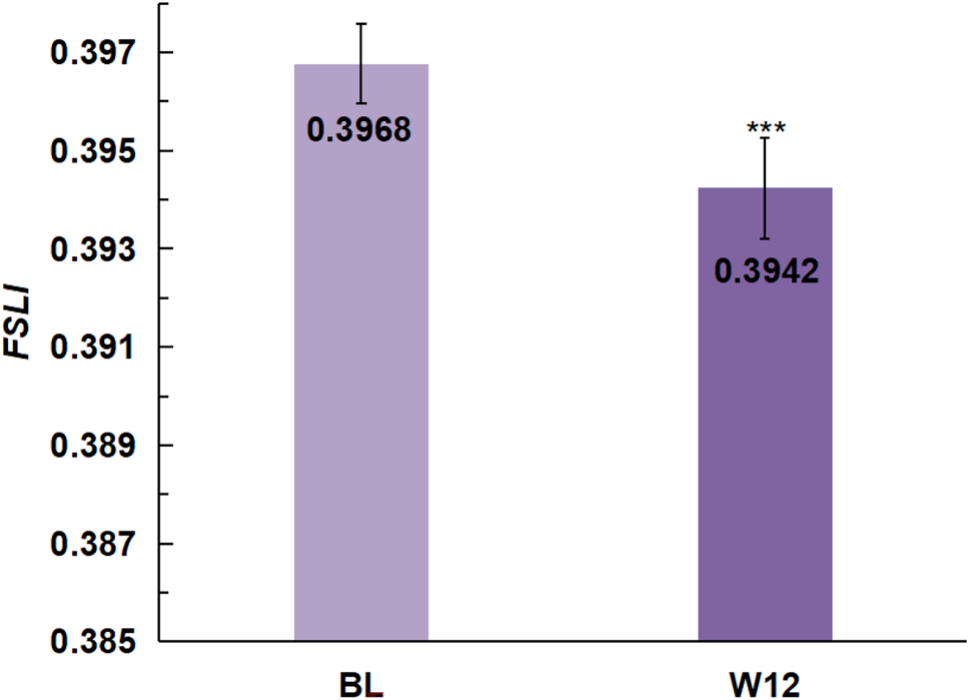
Change in 12-week *FSLI* for oral and topical beauty products.

*FSLI* was applied to evaluate the efficacy of oral cosmetic products combined with topical cosmetic products to improve the skin. In this example, we used parametric indicators from a previous clinical study. The study had been registered with the Chinese Clinical Trial Registry (registration No. ChiCTR2400085413, 07/04/2024) and had passed the ethical review by the Shanghai Ethics Committee for Clinical Research (approval No. SECCR/2024-46-01, 06/06/2024). In that study, 15 Chinese female participants used oral and topical beauty products consecutively to quantify the changes in facial skin laxity through metrics collection and data analysis. Higher values of the *FSLI* represent a higher degree of facial skin laxity. As can be seen in Fig. 8, *FSLI* significantly decreased after 12 weeks of using the test products compared to baseline (*p *= 0.007, normal distribution, paired sample t-test, α = 0.05, two-tailed).

**Fig. 9:**
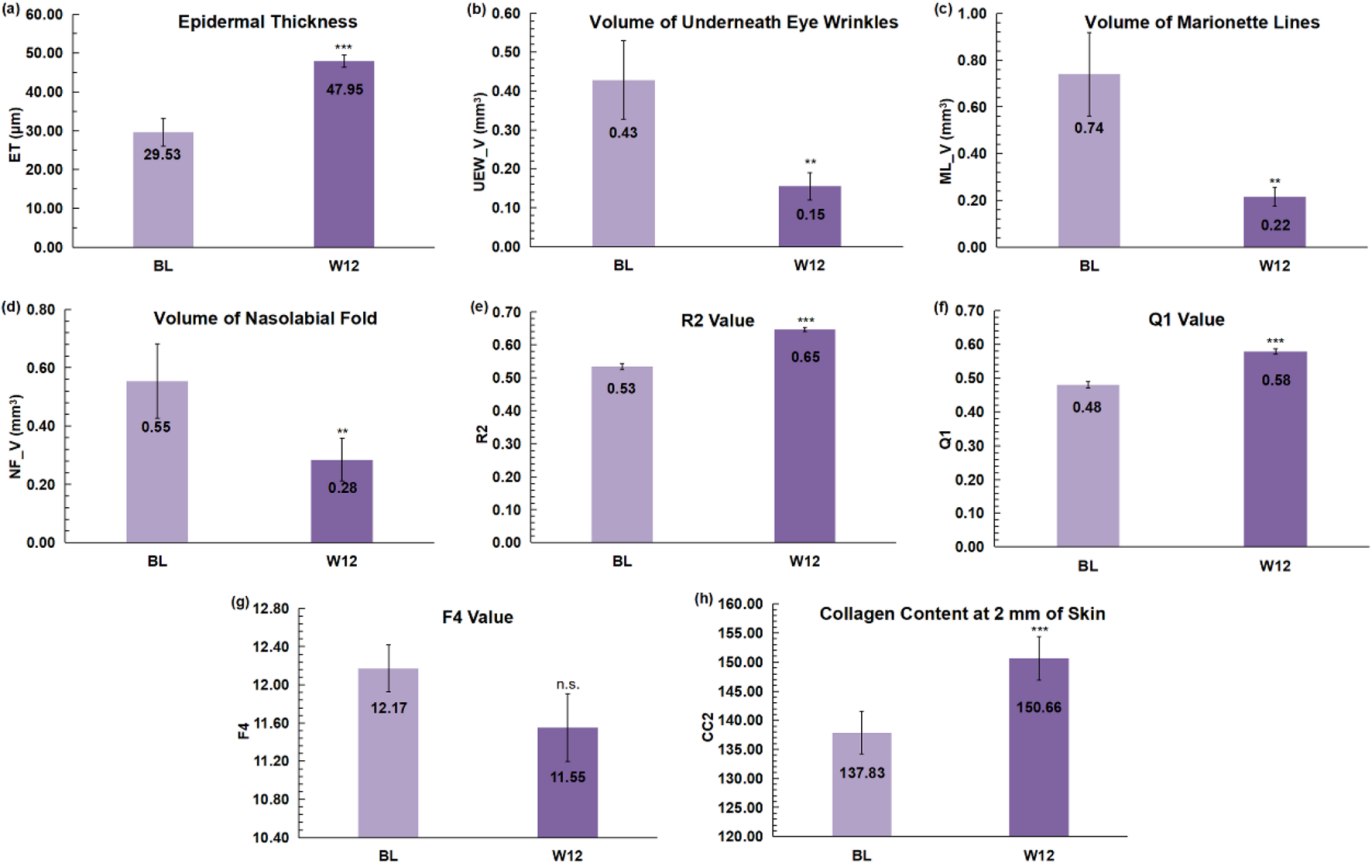
Change in 12-week non-invasive physiological parameters for oral and topical beauty products. *p < 0.05, **p < 0.01, ***p < 0.001, α = 0.05, two-tailed. (**a**), (**e**), (**f**), (**h**) Increases in ET, R2, Q1, and CC2 indicate that the skin condition has become better and tends to tighten; (**b**–**d**), (**g**) Decreases in UEW_V, ML_V, NF_V, and F4 indicate that the skin condition has become better and tends to tighten.

As shown in Fig. 9, analysis of non-invasive skin physiological parameters associated with the *FSLI* model showed that ET, R2, Q1, and CC2 were significantly improved at 12 weeks (*p *≤ 0.001, normal distribution, paired sample t-test, α = 0.05, two-tailed). UEW_V, ML_V, and NF_V were significantly decreased at 12 weeks (*p* = 0.001, non-normal distribution, Wilcoxon signed-rank test, α = 0.05, two-tailed). F4 improvement, although not significant (*p *= 0.064 > 0.05, normal distribution, paired sample t-test, α = 0.05, two-tailed), still tended to decrease. All of the above changes indicate that the skin condition has become better and tends to tighten.

**Fig. 10:**
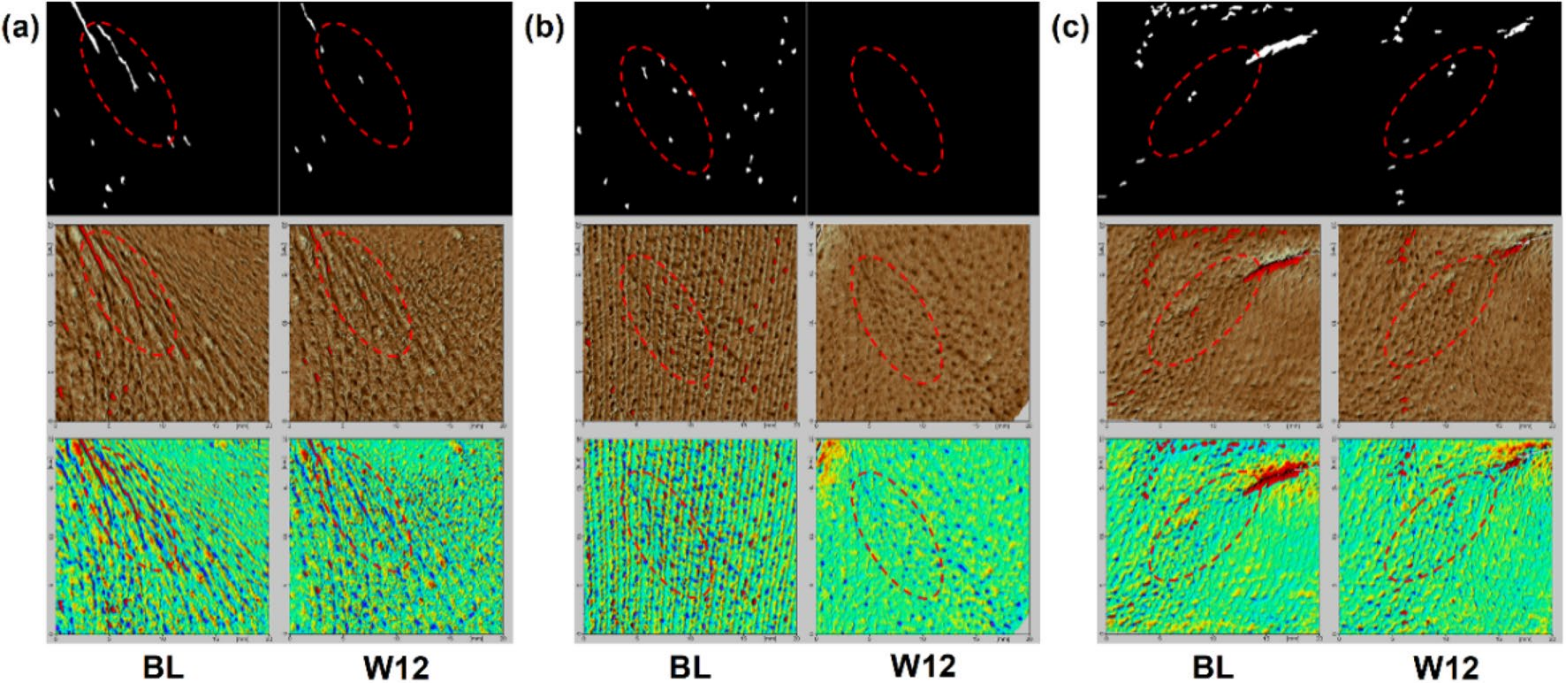
Significant lightening of wrinkles at 12 weeks. (**a**) Underneath eye wrinkles; (**b**) Nasolabial folds; (**c**) Marionette lines.

Significant lightening of underneath eye wrinkles, nasolabial folds, and marionette lines at 12 weeks as shown in Fig. 10.

## Discussion

Facial skin laxity is one of the more recognizable and perceptible facial conditions that Asians experience in their daily lives. Understanding how it is perceived and the underlying components of this phenomenon can help the beauty and skincare industry provide consumers with effective beauty management interventions. By examining this phenomenon through expert assessment, consumer perception, and noninvasive skin physiological measurements, we can achieve a more scientifically grounded characterization of aging skin. This approach facilitates the development of a daily skincare regimen that effectively addresses skin conditions. In this study, we assessed the apparent age of the participants’ skin, learned about the perception of facial skin laxity in the Chinese population, and measured relevant noninvasive skin physiological parameters. In addition, we investigated their joint effect by means of SVM regression modeling, which provided a key tool to quantitatively characterize skin clarity. Consistent with expectations, facial sagging was negatively correlated with skin elasticity R2 values and positively correlated with skin elasticity F4 values. When assigned ordinal ratings via the Bradley-Terry model, only 57 of the 64 participants received the appropriate rating, due to the fact that seven participants’ degree of facial collapse was always judged to be either low or high when compared. Therefore, when performing regression fitting with objective parameters, only data from 57 participants were modeled, which is a small sample size. Therefore, the validation method chosen in the regression modeling process of the support vector machine was the leave-one-out method, and the final model accuracy was 0.811, indicating that the model has good fitting ability.

However, it is important to acknowledge that the data in this study were derived from a previous human efficacy evaluation program with participants who self-identified as having trouble with skin laxity and who were screened for elasticity and wrinkle grades, which may have led to a decrease in the accuracy of the model in a population with mild or extremely slight laxity. In addition, the study mainly used subjective assessment and noninvasive skin physiological parameters, which may have some subjective bias. In conclusion, further longitudinal validation of the *FSLI *model over time or across product categories is still needed. Histologic correlation data support, such as collagen and elastin biopsies, may also be considered in future studies.

Given that the concept of loose facial skin is influenced by the cultural background of the perceiver, the model developed in this study only reflects the perceptions of Chinese or Asian individuals who have a similar appreciation for certain skin characteristics. If interest emerges in other parts of the world, other ethnicities’ perceptions of facial skin laxity should be studied and modeled. We hope that this study will provide a methodological framework for future global studies on modeling facial skin laxity.

## Conclusions

Based on the findings of this study, we conclude that facial skin laxity can be effectively characterized, quantified, and predicted using a linear model that incorporates objectively measured, noninvasive physiological parameters of the facial skin. This study contributes valuable data for both expert assessment and consumer-perceived evaluation of facial skin laxity, enabling the objective quantification of skin looseness through the non-invasive measurement of physiological skin parameters.

## Data Availability

The datasets used and analyzed during the current study are available from the corresponding author upon reasonable request.
